# Protracted Abstinence From Extended Cocaine Self-Administration Is Associated With Hypodopaminergic Activity in the VTA but Not in the SNc

**DOI:** 10.1093/ijnp/pyaa096

**Published:** 2020-12-11

**Authors:** Adélie Salin, Virginie Lardeux, Marcello Solinas, Pauline Belujon

**Affiliations:** Université de Poitiers, INSERM, Laboratoire de Neurosciences Expérimentales et Cliniques, Poitiers, France

**Keywords:** Cocaine, dopamine, substantia nigra pars compacta, ventral tegmental area, withdrawal

## Abstract

The chronic relapsing nature of cocaine addiction suggests that chronic cocaine exposure produces persistent neuroadaptations that may be temporally and regionally dynamic in brain areas such as the dopaminergic (DA) system. We have previously shown altered metabolism of DA-target structures, the ventral and dorsal striatum, between early and late abstinence. However, specific changes within the midbrain DA system were not investigated. Here, we investigated potential time- and region-specific changes of activity in the ventral tegmental area (VTA) and the substantia nigra pars compacta (SNc) in rats that had extended or limited access to cocaine and later underwent a period of abstinence. We found that DA activity is decreased only in the VTA in rats with extended access to cocaine, with no changes in SNc DA activity. These changes in VTA DA activity may participate in the negative emotional state and the incubation of drug seeking that occur during abstinence from cocaine.

## Introduction

Persistent risk of relapse to drug addiction even after protracted abstinence is believed to be associated with dynamic neuroadaptations that occur even after drug discontinuation (Grimm et al., 2001). In humans, chronic cocaine produces regional and temporal alterations in circuits involved in reward, motivation, and salience as well as stress reactivity and negative affect (Volkow et al., 2013). Cocaine addiction is associated with drug-induced neuroadaptations within the mesocorticolimbic dopaminergic (DA) system and its neural circuit projections to cortical and limbic structures (Nestler, 2002). For example, self-administration of cocaine has been shown to induce an increase of the α-amino-3-hydroxyl-5-methyl-4-isoxazolepropionate/N-methyl-D-aspartate ratio, lasting up to 3 months after the last injection (Chen et al., 2008) and suggesting an increased excitatory synaptic function in ventral tegmental area (VTA) DA neurons. Although addiction research has mainly studied alterations in the mesolimbic circuit, the nigrostriatal circuit could also contribute to the persistence of cocaine seeking (Volkow et al., 2006). A recent study has shown that the mesolimbic and nigrostriatal circuits seem to be differentially affected as abstinence progresses with a shift from ventral to more dorsal striatal regions (Nicolas et al., 2017). A decrease in DA levels in the nucleus accumbens (NAc) (Calipari et al., 2013), associated with a increase in reward threshold in rodents (Markou and Koob, 1991), has been described shortly after the last cocaine exposure, whereas cue-induced cocaine craving in humans is associated with increased DA transmission in the dorsal striatum (DSt) (Volkow et al., 2006). Although changes in synaptic plasticity at excitatory afferents in the VTA during early and late withdrawal have been extensively studied (Lüscher and Malenka, 2011), changes in spontaneous activity of DA neurons in the VTA and in the SNc have been less investigated, in particular during protracted abstinence. Therefore, in this study, using a well-established model of escalation of cocaine intake (Ahmed and Koob, 1998), we investigated longitudinal changes of DA activity in the VTA and the SNc after early and late abstinence from long exposure to chronic cocaine.

## Methods

### Animals

Adult male Sprague Dawley rats (8–10 weeks of age) (Janvier Labs, Le Genest-Saint-Isle, France) were housed in a temperature- and humidity-controlled environment and maintained on a 12-h-light/-dark cycle (on at 8:00 am). Rats were housed 2 per cage from arrival to surgery and 1 per cage after surgery. Rats had ad libitum access to food and water in their homecages. All experiments were conducted during the light phase as previously done (Nicolas et al., 2017) and in accordance with the European Union directives (2010/63/EU) for the care of laboratory animals, and all experimental protocols were approved by the local ethics committee (COMETHEA).

### Cocaine Self-Administration

#### Catheter Implantation

Rats were prepared for cocaine self-administration by surgical catheterization of the right jugular vein, as previously described (Nicolas et al., 2017). Rats were allowed to recover for 7 days and flushed for 4 days post-surgery with 0.1 mL sterile saline (0.9 %), gentamicin (20 mg/mL), and heparin (100 UI/mL) in sterile saline.

#### Apparatus

Experiments were conducted in operant-conditioning chambers equipped on a same wall with 2 retractable levers as operanda, a cue-light above the levers, and a house light. Chambers were controlled by Med Associates interface and software (Med Associates, St. Albans, VT).

#### Procedure

**Figure 1.**
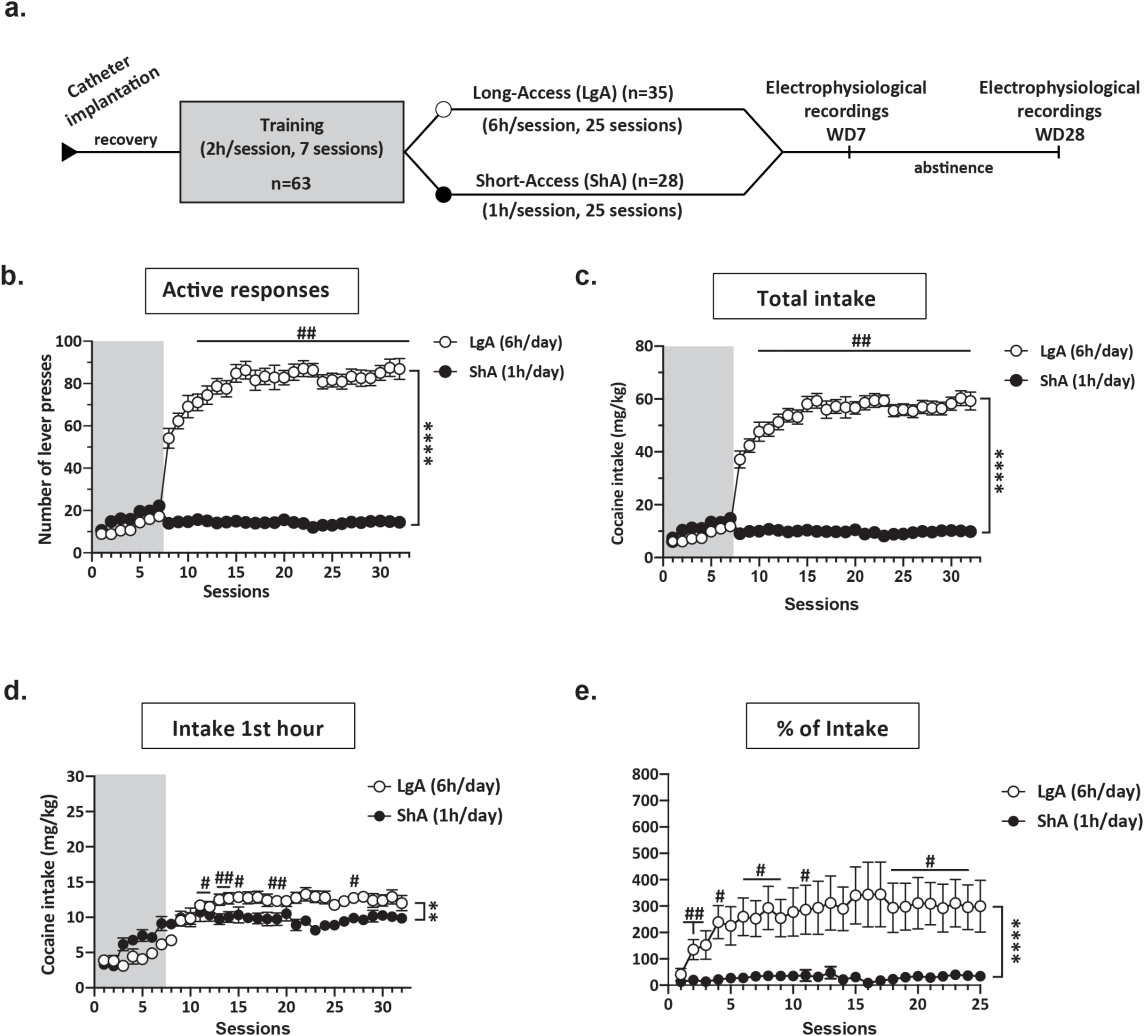
Cocaine self-administration. (A) Experimental design timeline. (B) Number of active lever presses during the training phase in which animals had access to cocaine for 2 hours and during the escalation phase in which rats were divided into short-access (ShA, n = 28, 1 hour sessions) and long-access (LgA, n = 35, 6 hours sessions) groups. (C) Total intake. (D) First hour intake. (E) Percentage of intake from last day of training. *P < .05, **P < .01, ****P < .0001. #*P* < .05, #*P* < .01, compared with the first day of self-administration session.

Rats were allowed to self-administer cocaine (Cooper, Bordeaux, France; 6 g/L in saline solution 0.9 %) according to a fixed ratio 1 of reinforcement. Response on the “active” lever resulted in the retraction of the levers, 1 i.v. cocaine infusion with the concomitant activation of the cue light that remained on for 5 seconds and was then pulsed for 5 seconds, followed by a 5-second time-out. Pressings on the “inactive” lever were recorded but had no consequences. Rats were first trained for seven 2-hour-daily sessions and then separated into 2 experimental groups, short access (ShA) and long access (LgA), to mimic recreational and addictive-like cocaine intake, respectively (Ahmed and Koob, 1998). Rats then had access to cocaine for 25 additional sessions for 1 h/d in the ShA group and 6 h/d in the LgA group. After the last self-administration session, rats remained in their homecages for up to 1 month of forced abstinence. Rats were then used for electrophysiological recordings after 1 week (WD7, between withdrawal days 6 and 9) or 1 month (WD28, between withdrawal days 28–38) of abstinence (Fig. 1A).

### In Vivo Single-Unit Extracellular Recordings

Rats were anesthetized with isoflurane (5% induction, 2% maintenance) and placed in a stereotaxic frame. Extracellular recording electrodes were pulled from glass micropipettes and filled with a 2% Chicago Sky Blue dye (Sigma-Aldrich, Saint-Quentin Fallavier, France) solution in 2 M NaCl.

**Figure 2.**
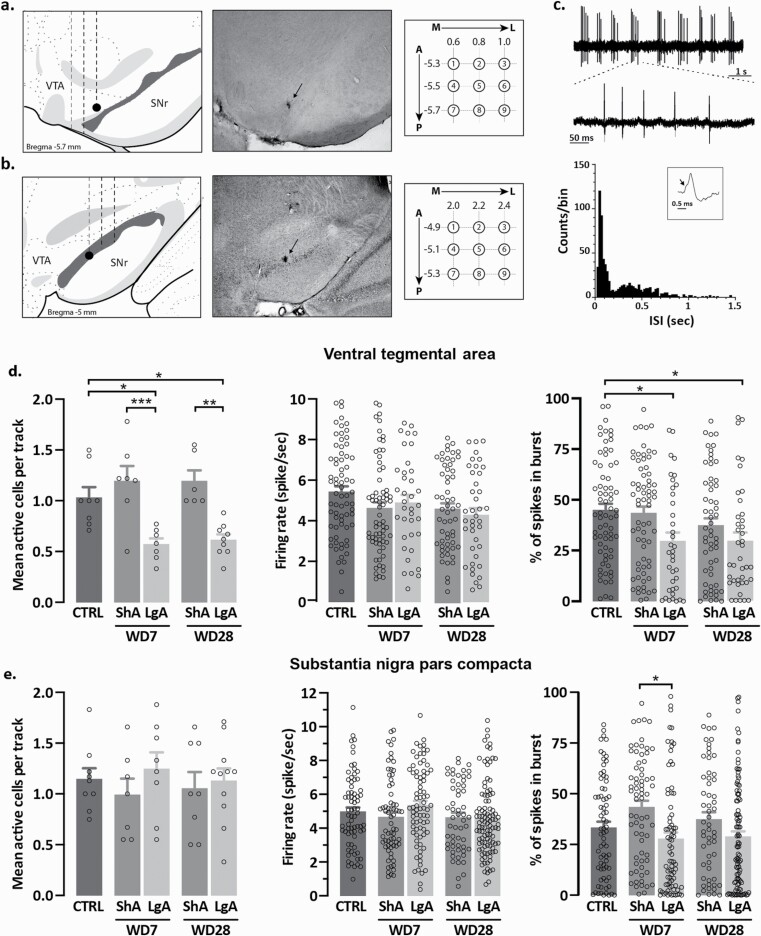
Chronic cocaine induces persistent decrease in ventral tegmental area (VTA), but not substantia nigra pars compacta (SNc), dopaminergic (DA) activity. Population activity in the VTA (A) and SNc (B) was measured in 9 electrode tracks separated by 0.2 mm (VTA  =  AP: 5.3–5.7 mm posterior from bregma, ML: 0.6–1.0 mm lateral from midline, and DV: 6.5–9.0 mm from the brain surface; SNc  =  AP: 4.9–5.3 mm posterior from bregma, ML: 2.0–2.4 mm lateral from midline, and DV: 6.5–9.0 mm from the brain surface). Corresponding histological slices show a Chicago sky blue deposit tracks in the VTA (A, middle) and SNc (B, middle) (black arrow). (C) Top, Representative traces from a VTA DA neuron displaying bursting activity. Inset represents a 500-msec close-up. Bottom, Representative histogram of interspike intervals (ISI) of a bursty VTA DA neurons. Inset, DA neurons display a biphasic (positive-negative) action potential, typically with a “notch” in the rising phase corresponding to the calcium-dependent initial segment spike (arrow) and a prominent negative component and with a total duration ≥2.2 msec. The duration from the spike initiation to the maximal negative phase of the action potential is ≥1.1 ms. (D) VTA DA neuron population activity (left), firing rate (middle), and percentage of spikes in bursts (right) in control (n = 8 rats, 70 neurons), short-access (ShA) (withdrawal day [WD] 7: n = 7 rats, 68 neurons; WD 28: n = 6 rats, 57 neurons) and long-access (LgA) (WD7: 7 rats, 37 neurons; WD28: n = 9 rats, 40 neurons) rats at WD7 and WD28. (E) SNc DA neuron population activity (left), firing rate (middle), and percentage of spikes in bursts (right) in control (n = 9 rats, 73 neurons), ShA (WD7: n = 7 rats, 69 neurons; WD28: n = 8 rats, 57 neurons) and LgA (WD 7: 8 rats, 78 neurons; WD28: n = 11 rats, 105 neurons) rats at WD7 and WD28. *P < .05, **P < .01, ***P < .001.

The recording electrode was lowered through the VTA or SNc in 9 sequential vertical “tracks” separated by 0.2 mm (Fig. 2A–B, right) (VTA: −5.3 to −5.7 mm posterior to bregma, 0.6 to 1.0 mm lateral to midline, and −6.5 to −9.5 mm ventral to the brain surface; SNc: −4.9 to −5.3 mm posterior to bregma, 2.0 to 2.4 mm lateral to midline, and−6.5 to −9.5 mm ventral to the brain surface), and all active cells encountered were recorded for 180 seconds to determine the average of active DA cells within each animal (cells/track, or population activity) (Belujon et al., 2016; Bortz and Grace, 2018). Neurons were identified as DA using well-established criteria (Ungless and Grace, 2012). Single-unit activity recorded from the VTA and the SNc was amplified 10 times, filtered (low pass: 30 Hz; high pass: 16 kHz), and further amplified 50 times (Multiclamp 700b, Axon Instruments, Union City, CA). The signal was digitized at 16 kHz (CED 1401) and acquired on a computer using Spike 2 7.0 software (Cambridge Electronics Design, Cambridge, UK). The following electrophysiological parameters were analyzed: basal firing rate, bursting rate, and the percentage of spikes in bursts.

### Histology

Electrode placements were verified via electrophoretic ejection of Chicago Sky Blue dye (Sigma Aldrich) at the more ventral recording site of the last track. Rats were killed with a lethal dose of pentobarbital (>150 mg/kg, i.p.) and brains were removed. The tissue was fixed in 8% paraformaldehyde for at least 48 hours and transferred to a 25% sucrose solution for cryoprotection. Once saturated, the brains were frozen and sliced coronally at 60 μm thick using a cryostat (Thermo Scientific Cryostat CryoStar NX70) and mounted onto gelatin-coated slides. Tissue was stained with a combination of neutral red and cresyl violet. Only rats with verified electrode placements were included in the data analysis.

### Statistical Analysis

For self-administration, 2-way repeated-measures ANOVA (or a mixed model when values were missing) with time as a within-subject factor and cocaine exposure (LgA or ShA) as a between subject factor was used. Results showing significant overall changes were subjected to a Sidak post hoc test.

For electrophysiological experiments, a 1-way ANOVA was used. Results showing significant results were subjected to a Tukey post hoc test.

## Results

### Self-Administration

Long-access rats showed an increase in the number of active lever presses (Fig. 1B) and cocaine intake (Fig. 1C), whereas ShA rats showed stable self-administration over time (active press: time: F_(24,1464)_ = 5.906, *P* < .0001; group: F_(1,61)_ = 359.3, *P* < .0001; group × time interaction: F_(24,1464)_ = 6.248, *P* < .0001; intake: time: F_(24,1464)_ = 6.142, *P* < .0001; group: F_(1,61)_ = 359.3, *P* < .0001; group × time interaction: F_(24,1464)_ = 25.92, *P* < .0001). During the first hour of access to the drug (Fig. 1D), ShA rats showed stable intake, whereas LgA showed an escalation of their intake over time (time: F_(24,1418)_ =  3.126, *P* < .01; group: F_(1,62)_ = 6.018, *P* < .05; group × time interaction: F_(24,1418)_ = 6.079, *P* < .0001). Figure 1e shows the mean percentage change of intake, normalized to the first hour of the last session of training, revealing an escalation of intake in LgA rats compared with ShA rats (time: F_(24,1418)_ = 3.439, *P* < .05; group: F_(1,62)_ = 8.767, *P* < .01; group × time interaction: F_(24,1418)_ = 2.740, *P* < .0001).

### Abstinence From Cocaine Intake Induces Changes in Dopaminergic Activity in the VTA but Not in the SNc

Locations of recording electrodes are presented in Figure 2A–B. Bursting activity (Fig. 2c), firing rate, and population activity were analyzed for VTA and SNc DA neurons. Extended exposure to cocaine (LgA rats) induced a significant decrease in population activity of VTA DA neurons during abstinence both at short-term (WD7) and long-term (WD28) abstinence compared with control and ShA rats (1-way ANOVA, F_(4,32)_ = 9.915, *P* < .0001, Tukey’s post hoc) (Fig. 2D, left). Although there was no change in the firing rate (1-way ANOVA, F_(4,267)_ = 2.125, *P* = .0781, Tukey’s post hoc; Fig. 2D, middle), in the VTA, LgA rats displayed a significant decrease in percentage of spike firing in bursts of DA neurons at WD7 and WD28 compared with control animals (1-way ANOVA, F_(4,267)_ = 4.044, *P* < .01, Tukey’s post hoc; Fig. 2D, right).

In the SNc, there was no change in the population activity (1-way ANOVA, F_(4,38)_ = 0.4151, *P* = .7967, Tukey’s post hoc; Fig. 2E, left) and the firing rate (1-way ANOVA, F_(4,377)_ = 1.641, *P* = .1633, Tukey’s post hoc; Fig. 2E, middle). LgA rats displayed a decreased bursting activity at WD7 compared with ShA rats (1-way ANOVA, F_(4,377)_ = 4.500, *P* < .01, Tukey’s post hoc; Fig. 2E, right).

## Discussion

The present study demonstrates that extended exposure to cocaine, followed by abstinence, induces a persistent decrease in DA activity in the VTA but no change in the SNc. Withdrawal after chronic cocaine impairs the activity of brain reward circuitry, which may contribute to affective changes during abstinence. For cocaine addiction, a negative affective state is mainly described during early withdrawal, but it disappears after long-term abstinence (Becker et al., 2017). A decrease in VTA DA activity plays a pivotal role in hedonic deficits (Belujon et al., 2016) and could be responsible for the negative state observed early during abstinence. Persistent plastic changes in the VTA have been previously described during abstinence. In particular, an increase in the α-amino-3-hydroxyl-5-methyl-4-isoxazolepropionate/N-methyl-D-aspartate ratio, which reflects an increase in synaptic excitatory inputs to the VTA, has been observed following early (1 day) and late (45 days) abstinence from cocaine self-administration (Chen et al., 2008). Here, we show in vivo a persistent decrease in population activity and bursting activity in the VTA. This reduction is consistent with a previous study showing long-term reduction of population activity after repeated cocaine injections (Shen et al., 2007). The reduction in the number of active VTA DA neurons could be caused by overexcitation to the extent of depolarization block (Shen et al., 2007). To our knowledge, there are no data on SNc spontaneous activity, or plastic changes in the SNc, under similar conditions (i.e., after cocaine self-administration). SNc and VTA DA neurons have different pacemaking mechanisms (Morikawa and Paladini, 2011). However, since we found no significant changes in firing rate in both structures, it is unlikely that the difference in the activity of VTA and SNc DA neurons comes from intrinsic properties of firing.

Modulation of the DA system by specific afferents plays a key role in different pathology states, including addictive behaviors (Belujon et al., 2016; Belujon and Grace, 2017). In particular, the basolateral amygdala (BLA) has been consistently shown to be responsible for the decrease in VTA DA population activity (Chang and Grace, 2013; Belujon et al., 2016), and therefore, persistent changes of specific afferents to the VTA could be responsible for the decrease in VTA population activity. VTA DA population activity is modulated by 2 distinct circuits, an activating circuit, involving the ventral subiculum of the hippocampus, and an inhibiting circuit, involving the BLA (Belujon and Grace, 2017). Therefore, cocaine-induced decreases in DA activity found in this study may originate from dysregulation in the BLA. In agreement with this hypothesis after long-term abstinence from cocaine, spontaneous firing rate and the number of spontaneously active neurons per track in the BLA have been shown to be increased in rats that had extended access to cocaine, indicating an overall increase in BLA population activity following cocaine treatment (Munshi et al., 2019; Salin and Belujon, unpublished results). Since SNc DA neuron spontaneous activity is not altered during long-term abstinence, it is possible that potential cocaine-induced changes in afferent structures modulating SNc DA population activity are not sufficient to induce persistent modifications.

Whereas early cocaine withdrawal is associated with a negative affective state, an incubation of craving for the drug has been described after long-term abstinence (Grimm et al., 2001), suggesting neuroadaptations occurring during abstinence. Importantly, increases in cocaine craving has been described as soon as 1 week after discontinuation of cocaine self-administration, but it reaches its peak after 30–90 days of abstinence (Grimm et al., 2001). Several withdrawal-dependent neuroadaptations in the NAc, VTA, and related circuits have been described (Loweth et al., 2014; Li et al., 2015; Wolf, 2016). In the present study, the decrease in the VTA DA activity started early and lasted up to 1 month of abstinence, suggesting that the behavioral changes that only appear after a long abstinence might not be a direct consequence of persistent changes in the VTA but rather of synaptic changes occurring in downstream regions such as the medial prefrontal cortex (mPFC) and the NAc (Creed and Lüscher, 2013). Thus, incubation of cocaine craving is accompanied by increased recruitment of NAc neurons time-locked to drug-related cues (Hollander and Carelli, 2007) and remodeled mPFC and BLA afferents to the NAc (Dong, 2016). Low VTA spontaneous activity of DA neurons after long periods of abstinence may also induce a glutamate–DA imbalance in downstream regions, such as the NAc or the mPFC that would increase sensitivity to cue, leading to increased craving. In the NAc, some studies report reduced electrically evoked dopamine (Calipari et al., 2013) while others report an increase (Cameron et al., 2016). In the ventromedial PFC, incubation of cocaine craving is associated with an increased glutamate release concomitant with a relative decrease in DA release (Shin et al., 2016). These studies suggest a time-dependent dysregulation of the glutamate–DA balance within DA target structures that may participate in incubation of cocaine craving.

In the present study, we found no significant deficit in SNc DA activity in rats that had extended access to cocaine whether after early or late abstinence. However, clinical studies have shown an increased DA release in the DSt, which was correlated with self-reports of craving (Volkow et al., 2006). Moreover, preclinical studies have shown that cocaine-induced plastic changes from specific excitatory afferents to the SNc (Beaudoin et al., 2018) and that after long-term abstinence, the activity of the dorsal rather than the ventral striatum is altered (Nicolas et al, 2017). It is therefore possible that synaptic plastic changes in the SNc do not induce changes in spontaneous activity of DA neurons and that changes in DA release in the DSt originated from dysregulation of DA release in target structures. Indeed, DA release is differently modulated in the NAc and in the DSt (Shin et al., 2017). Therefore, it is possible that cocaine affects DA release in the DSt even though chronic cocaine has no direct effect on DA population activity in the SNc.

A limitation of the current study is that DA activity in the VTA and the SNc was measured only in male rats. Incubation of cocaine craving has been shown to be higher in female rats compared with male rats after intermittent access to cocaine (Nicolas et al., 2019). However, in the present study, rats had continuous access to cocaine, a schedule that was not associated with differences between males and females (Nicolas et al., 2019). Further studies are, however, required to determine whether a similar decrease in DA VTA activity occurs in female rats.

In conclusion, in this study we found that escalation of cocaine self-administration produced short- and long-term decreases in the spontaneous activity of DA neurons in the VTA but not the SNc. In the early stages of abstinence, this decrease may be responsible for the psychological withdrawal symptoms experienced by cocaine addicts. On the other hand, in later stages of withdrawal, in response to relevant drug-related stimuli, this decrease may lead to neurotransmitter imbalance in projection structures, leading to increased craving and risks of relapse.
